# Sympathetic Ophthalmia

**Published:** 1899-12-23

**Authors:** 


### SYMPATHETIC OPHTHALMIA.

In a post graduate lecture on wounds of the ciliary region and their treatment, recently delivered at the West London Hospital, Mr. Percy Dunn insisted on the relation between sympathetic ophthalmia and the presence of some pathogenic organism, and pointed out that before Listerism became part of the professional creed of surgeons sympathetic ophthalmia was of frequent occurrence, whereas since antiseptics had been employed in eye surgery the disease had become more and more infrequent, until nowadays it might with truth be described as an ocular pathological curiosity. This had given strong support to the belief that the disease owed its origin to a pathogenic organism. What particular organism was engaged in the process he could not say, but he protested again against the view that the non-discovery of any special germ of this disease should be held to militate against the idea that such a germ was involved in its causation. To argue that a germ which could not be inoculated, cultivated, or stained by the methods now known could not possibly have any existence, must, he said, obviously be wrong, for it would imply that bacteriology had already reached a position of finality. The reasonable position to take up in regard to these cases was that the technique of bacteriology was at fault; and he said that, taking into consideration all that was known on the subject, he considered that we could not be far wrong in assuming that sympathetic ophthalmia was a microbic disease, that as such it was preventable, and that the secret of its prevention was the strict observance of aseptic and antiseptic principles in eye surgery.

